# Effects of enrichment strategies on outcome of adrecizumab treatment in septic shock: *Post-hoc* analyses of the phase II adrenomedullin and outcome in septic shock 2 trial

**DOI:** 10.3389/fmed.2022.1058235

**Published:** 2022-12-01

**Authors:** Dirk van Lier, Adrien Picod, Gernot Marx, Pierre-François Laterre, Oliver Hartmann, Claudia Knothe, Feriel Azibani, Joachim Struck, Karine Santos, Jens Zimmerman, Andreas Bergmann, Alexandre Mebazaa, Peter Pickkers

**Affiliations:** ^1^Department of Intensive Care Medicine and Radboud Center for Infectious Diseases (RCI), Radboud University Medical Center, Nijmegen, Netherlands; ^2^Anesthésie-Réanimation, Hôpital Lariboisière, AP–HP, Paris, France; ^3^Klinik fur Operative Intensivmedizin und Intermediate Care, Universitätsklinikum Aachen, Aachen, Germany; ^4^Unité de Soins Intensifs, Cliniques Universitaires Saint-Luc (UCL Bruxelles), Brussels, Belgium; ^5^Sphingotec GmbH, Hennigsdorf, Germany; ^6^Adrenomed AG, Hennigsdorf, Germany; ^7^4TEEN4 Pharmaceuticals GmbH, Hennigsdorf, Germany; ^8^Université de Paris, U942 Inserm, MASCOT, APHP, Fédération Hospitalo-Universitaire PROMICE, Hôpitaux Universitaires Saint-Louis-Lariboisière, Paris, France

**Keywords:** sepsis, septic shock, adrenomedullin, dipeptidyl peptidase 3, AdrenOSS-2, Adrecizumab

## Abstract

**Purpose:**

Adrecizumab, a non-neutralizing antibody of adrenomedullin (ADM) was recently investigated regarding its potential to restore endothelial barrier function in septic shock patients with high plasma ADM levels. Circulating dipeptidyl peptidase 3 (cDPP3), a protease involved in the degradation of several cardiovascular mediators, represents another biological pathway strongly associated with outcome in septic shock, although unrelated to ADM. Therefore, the prognosis of patients with elevated cDPP3 may not be influenced by Adrecizumab. Also, time until initiation of treatment may influence efficacy.

**Objective:**

To evaluate effects of cDPP3-based enrichment on treatment efficacy of Adrecizumab.

**Materials and Methods:**

*Post-hoc* analysis of AdrenOSS-2, a phase-II, double-blind, randomized, placebo-controlled biomarker-guided trial of Adrecizumab.

**Results:**

Compared to the total study cohort [HR for 28-day mortality of 0.84 (95% CI 0.53;1.31), *p* = 0.439], therapeutic benefit of Adrecizumab tended to be more pronounced in the subgroup of 249 patients with low cDPP3 (<50 ng/mL); [HR of 0.61 (95% CI 0.34;1.08), *p* = 0.085]. Median duration to study drug infusion was 8.5 h. In the subgroup of 129 patients with cDPP3 <50 ng/mL and an early start of treatment (<8.5 h after septic shock diagnosis) HR for 28-day mortality vs. placebo was 0.49 (95% CI 0.21–1.18), *p* = 0.105. In multivariate interaction analyses corrected for baseline disease severity, both cDPP3, as well as the cDPP3 * treatment interaction term were associated with a reduced HR for 28-day mortality in the Adrecizumab treated group; *p* = 0.015 for cDPP3 in univariate analysis, *p* = 0.025 for the interaction term between cDPP3 and treatment group. In contrast, treatment timing was not significantly associated with 28-day mortality in multivariate interaction analyses.

**Discussion:**

In septic shock patients with high ADM levels, a further *post-hoc* enrichment strategy based on cDPP3 may indicate (with all the caveats to be considered for *post-hoc* subgroup analyses) that therapeutic efficacy is most pronounced in patients with lower cDPP3 levels.

## Introduction

Sepsis is defined as an inflammatory disorder, during which a dysregulated host response to infection results in life threatening organ dysfunction, leading to substantial patient mortality (1). During sepsis, both vascular tone and vascular integrity are compromised, ultimately contributing to the development of septic shock (2). Septic shock is characterized by increased blood lactate levels, as well as the necessity for vasopressor therapy to maintain mean arterial pressure despite adequate fluid resuscitation (1, 3). Although knowledge on the molecular mechanisms associated with sepsis development has vastly increased, treatment strategies remain virtually unchanged, consisting of supporting therapies including adequate and timely antimicrobial therapy, control of infection, fluid resuscitation and vasopressor therapy to maintain vascular tone (4).

Adrenomedullin (ADM) is a hormone essential for regulation of endothelial barrier function (5). During sepsis, multiple processes stimulate ADM release (6, 7) and increased ADM levels are associated with sepsis severity, development of organ dysfunction, including vasopressor/inotrope dependency (8–10), as well as mortality. In preclinical sepsis models, Adrecizumab, a non-neutralizing monoclonal anti-ADM antibody, improved endothelial barrier function and reduced organ failure, as well as mortality (11–13). Following the results of these preclinical studies, dose-finding, as well as a proof-of-mechanism in healthy volunteers (14), the proof-of-concept phase-2 trial AdrenOSS-2 was performed to evaluate the safety, tolerability and efficacy of Adrecizumab in septic shock patients (10, 15).

An increasing body of evidence demonstrates that multiple signaling pathways are altered during sepsis (16), reinforcing the notion that sepsis represents a highly heterologous syndrome (17). In AdrenOSS-2 the baseline concentration of the molecular biomarker “biologically active Adrenomedullin” (bio-ADM) was implemented as an enrichment strategy, aimed at reducing patient-heterogeneity by selecting patients with a higher mortality risk and (based on the mechanism of action) improved chances of treatment benefit. While this enrichment strategy represents one of the first examples of personalized medicine in sepsis trial design, significant further reductions in patient heterogeneity might be achieved by combining multiple biomarkers for patient selection.

Dipeptidyl peptidase 3 (DPP3) is an ubiquitous catalytic enzyme only present at low plasma concentration in healthy subjects, where it is involved in the degradation of several important regulators of vascular tone (18, 19). During septic shock, high concentrations of circulating DPP3 (cDPP3 for circulating DPP3) are associated with impaired outcome (20). Putatively, high concentrations of cDPP3 are the result of increased release of DPP3 caused by cellular injury (19). Interestingly, preclinical animal studies demonstrated that intravenous injection of DPP3 in healthy rodents caused impairment of cardiac function, while cDPP3 inhibition with a specific monoclonal antibody restored cardiac function in a rodent sepsis model (21). Based on these findings, DPP3 appears to represent a distinct molecular pathway in septic shock (19). This pathway is most likely contributing to the outcome of septic shock in a way not directly related to adrenomedullin.

We hypothesize that a subset of septic shock patients with high bio-ADM levels also presents with high cDPP3 levels. Moreover, we hypothesize that patients with high cDPP3 levels have worse outcome, as well as a less pronounced therapeutic benefit when treated with Adrecizumab, since increased cDPP3 represents an independent risk factor of death in septic shock that is not targeted by Adrecizumab. Also, as it is generally recognized that early initiation of treatment may improve clinical outcome in septic shock patients, subgroup analyses based on time between diagnosis of septic shock and start of treatment were also conducted, both with and without stratification based on cDPP3 levels.

## Materials and methods

### Study design

The AdrenOSS-2 (Adrenomedullin and Outcome in Septic Shock 2) trial was a double-blind, placebo-controlled, randomized, multicenter, proof-of-concept, biomarker-guided and dose-finding phase II trial (clinicaltrials.gov identifier NCT 02338843) (15). In brief, adults (≥18 years) in the early phase of septic shock could be enrolled. As this was a biomarker-guided trial, one inclusion criterion was an elevated bio-ADM plasma concentration at baseline (>70 pg/mL), a cut-off value based on the increased risk of impaired outcome found in previous studies in sepsis and septic shock patients (8–10, 22–24). Patients also needed to be in the early phase of septic shock, defined as start of vasopressor therapy <12 h before study inclusion.

Patients were randomly assigned in a 1:1:2 ratio to either Adrecizumab (HAM 8101) 2 mg/kg, 4 mg/kg bodyweight or placebo. Patients received the assigned trial medication as a single intravenous infusion, administered over approximately 1 h. Dose selection was based on the phase-I study results (14), that showed a relevant Adrecizumab-induced elevation of plasma bio-ADM (over the bio-ADM concentrations present in the absence of Adrecizumab) for a time period of ∼7 days, the clinically most relevant phase in septic shock. Since Adrecizumab was given in molar excess of several hundred-fold above the concentration of bio-ADM in both treatment arms (2 mg/kg and 4 mg/kg), both dosage groups were pooled for the current *post-hoc* analysis.

### Measurements

Ethylenediaminetetraacetic acid (EDTA)-anticoagulated blood samples for determination of bio-ADM were obtained prior to randomization. As bio-ADM cut-offs served as one of the main inclusion criteria in the original AdrenOSS-2 protocol, a rapid measurement assay was used (8). For determination of cDPP3, EDTA-anticoagulated blood samples were taken immediately before start of administration of the investigational product. Blood was centrifuged for 10 min at 4°C, after which plasma was stored at −80°C until blinded analysis using a cDPP3 luminescence immunoassays (4TEEN4 Pharmaceuticals GmbH, Berlin, Germany). The details and design principles of this assay are provided elsewhere (25).

### Outcome evaluation

The main objective of this *post-hoc* analysis was to assess the interplay between cDPP3 concentrations and the therapeutic efficacy of Adrecizumab, as determined by changes in hazard ratios for 28-day mortality based on cDPP3 stratification. Secondly, we investigated the influence of cohort stratification based on time from diagnosis of septic shock to initiation of the study drug on 28-day mortality hazard ratios. Lastly, we assessed the influence of cohort stratification combining cDPP3 assessment and time until initiation of treatment on 28-day mortality hazard ratios. Secondary objectives of these *post-hoc* analyses included changes in SOFA score after 24 h of treatment, based on the aforementioned cohort stratification approaches. Safety endpoints included adverse event report rates in groups stratified by cDPP3 levels.

### Statistics

Continuous variables are presented as median [interquartile range (IQR)], whereas categorical variables are presented as counts and percentages. All reported outcomes are calculated for subgroups of the original intention to treat (ITT) study population. High cDPP3 levels were defined as ≥ 50 ng/mL for this *post-hoc* analysis. This cut-off signifies a concentration of cDPP3 well above the median of the AdrenOSS-2 ITT study, is outside the range of normal values determined for cDPP3 in earlier studies (19, 20, 25) and does not exclude a major subset of patients. For the comparison of “early” and “late” start of Adrecizumab treatment, cohorts were dichotomized at the median time from diagnosis of septic shock until start of treatment.

All-cause mortality for 28-day follow-up was evaluated and Kaplan–Meier plots were generated comparing Adrecizumab (combined doses) vs. placebo. The log-rank test was chosen for showing differences in mortality rates among treatment groups. In order to further investigate the interplay between cDPP3 concentration, treatment timing and therapeutic efficacy of Adrecizumab, we performed an interaction analysis between Adrecizumab treatment and baseline cDPP3 concentration, i.e., a Cox regression was performed that included cDPP3, treatment status and the interaction term. The same analysis was performed for time from diagnosis of septic shock until start of treatment instead of cDPP3. Both models also included baseline SOFA scores as a correction for baseline disease severity. For these interaction analyses, continuous variables were log-transformed. Proportional hazard assumptions of the generated models were tested by calculating the Schoenfeld residuals for all relevant covariates. We did not apply model adjustment for additional covariates (e.g., other candidate biomarkers), as the studies sample size was not sufficient, and thus underpowered to allow for the performance of multivariate regression analyses of this complexity.

For comparison of patient characteristics, the Kruskal–Wallis test was applied to continuous variables. Categorical variables are summarized category-wise with numbers and percentages and compared using the Chi^2^ test for contingency tables. Student’s *t*-tests were applied for comparisons (combined doses of Adrecizumab vs. placebo) of change in SOFA scores from baseline after 24 h of treatment. The primary aim of these *post-hoc* analyses is to determine the effect size for Adrecizumab employing further enrichment criteria. These analyses are exploratory, and smaller sample sizes of subgroups defined by further enrichment criteria need to be taken into account in the interpretation of the results. Based on decreasing sample size, it is anticipated that results of subgroups may not differ significantly, but may indicate trends of treatment effects. No correction for multiple testing was performed and a two-sided *p*-value of <0.05 is considered to indicate statistical significance. All analyses were performed using SAS version 9.3, and R version 3.4.3.^1^

## Results

### Study population and *post-hoc* subgroup analyses

A total of 459 patients were screened of which 301 were randomized to either placebo (*n* = 152) or Adrecizumab (*n* = 149) (15). The full list of inclusion and exclusion criteria can be found in the AdrenOSS-2 study protocol (15). Of all patients originally studied in AdrenOSS-2, plasma samples suitable for analysis of cDPP3 were missing in 3 patients, these patients were excluded from further analyses. Correspondingly, a total of 298 patients were available for these *post-hoc* analyses.

Median [IQR] age of the patients was 71.0 [61; 77] years, 61.1% of patients were male, median [IQR] APACHE-II score was 32 [29; 36], while the SOFA score was 10 [8; 12]. Sources of infection were predominantly abdomen (21.6%), lung (20.9%) or urinary tract (17.9%).

### Circulating dipeptidyl peptidase 3 levels and associations with admission characteristics and mortality (intention to treat population)

Baseline median [IQR] cDPP3 of the total cohort was 22.8 [14.6; 39.8] ng/mL. A total of 49 (16%) patients presented with cDPP3 levels above the specified cut-off of >50 ng/mL. The 28-day mortality rate of patients with a cDPP3 level >50 ng/mL was 51.0% (all treatment groups, *n* = 49) compared to 20.5% in the low cDPP3 group (*n* = 249). A more detailed analysis for subgroups with a cut-off level of cDPP3 of 50 ng/mL was applied. The patient subgroups split by this cut-off level exhibited important differences in sepsis severity at baseline, with SOFA score, inflammation related parameters like IL-6 and PCT, lactate concentrations, fluid input and norepinephrine dose requirements that were markedly higher in patients with a cDPP3 concentration of >50 ng/mL at baseline (Table 1). In both the low and high cDPP3 subgroups, no significant differences in baseline characteristics were observed between the Adrecizumab and placebo group, suggesting that randomization remained intact in these subgroups (Supplementary Table 1).

**TABLE 1:** Demographics and baseline characteristics.

	cDPP3 < 50 ng/mL (*n* = 249)	cDPP3 > 50 ng/mL (*n* = 49)	*P*-value
**Demographics**			
Age (years)	71 [62–78]	68 [61–75]	0.164
BMI (kg/m2)	25.8 [23.4–30.2]	26.0 [23.9–28.1]	0.674
Gender, female, *n* (%)	98 (39.4)	19 (38.8)	0.939
**Severity scores**			
SOFA (points)	9 [8–11]	11 [9–14]	<0.001
APACHE II (points)	32 [29–36]	32 [27–36]	0.594
**Hospital admission characteristics**			
Temperature (°C)	37.0 [36.4–37.6]	36.9 [36.4–37.4]	0.759
HR (bpm)	97 [83–111]	101 [88–121]	0.098
MAP (mmHg)	72 [66–79]	71 [65–77]	0.424
Norepinephrine requirement (mcg/kg/min)	0.411 [0.205–0.779]	0.760 [0.357–1.400]	<0.001
Mechanical ventilation, *n* (%)	131 (52.3)	32 (65.3)	0.103
IL-6 (pg/mL)	2016 [480–11112]	12055 [2657–53060]	<0.001
PCT (ng/mL)	37.88 [8.26–87.10]	62.72 [27.92–129.80]	0.005
eGFR, MDRD (ml/min*1.73 m^2^)	37.4 [22.7–58.0]	30.7 [21.4–38.0]	0.025
Lactate (mmol/L)	2.8 [1.6–4.6]	5.0 [3.7–8.3]	<0.001
PaO2/FiO2 (mmHg/%)	245 [176–343]	213 [136–343]	0.329
Fluid Input, first 24 h (mL)	2592 [1588–4274]	4044 [2894–4890]	<0.001
Time from septic shock to trt start (h)	8.4 [5.8–10.8]	9.5 [6.1–11.2]	0.224
**Origin of sepsis**			0.010
Lung, *n* (%)	53 (21.3)	9 (18.4)	
Peritonitis, *n* (%)	58 (23.3)	7 (14.3)	
Skin and soft tissue, *n* (%)	20 (8.0)	3 (6.1)	
Urinary tract, *n* (%)	49 (19.7)	4 (8.2)	
Other, *n* (%)	69 (27.8)	26 (53.1)	

Differences in continuous variables were assessed with Kruskal–Wallis tests, while differences in categorical variables were assessed using Chi^2^ tests.

cDPP3, circulating dipeptidyl peptidase 3; BMI, body mass index; SOFA, sequential organ failure assessment; APACHE, acute physiology and chronic health evaluation; HR, heart rate; MAP, mean arterial pressure; PCT, procalcitonin; eGFR, estimated glomerular filtration rate.

### Adrecizumab therapeutic efficacy by baseline circulating dipeptidyl peptidase 3 levels

**FIGURE 1:**
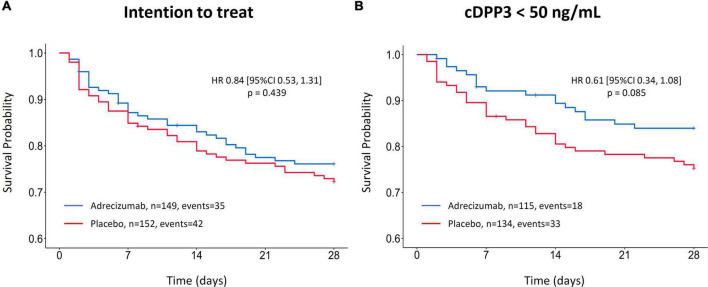
Kaplan–Meier of 28-day mortality after Adrecizumab/placebo infusion, based on circulating dipeptidyl peptidase 3 (cDPP3) stratification. Data is displayed for the original intention to treat (ITT) analysis (15) **(A)** and the cDPP3 <50 ng/mL subgroup **(B)**. Both Adrecizumab dosing groups were combined. Crosses represent censored patients.

In the original intention to treat (ITT) analysis (*n* = 301), the hazard ratio (HR) for 28-day mortality associated with Adrecizumab treatment was 0.84 (95% CI 0.53–1.31; *p* = 0.439; (Figure 1A). Corresponding incidence of 28-day mortality was 23 vs. 28%, *p* = 0.410 for the Adrecizumab and placebo groups, respectively. The significant interaction between cDPP3 concentration and Adrecizumab therapeutic efficacy was first illustrated by employing a cDPP3 level of 50 ng/mL as a cut-off. In patients with cDPP3 levels <50 ng/mL (*n* = 249) the HR for mortality associated with Adrecizumab tended to improve to 0.61 (95% CI 0.34–1.08; *p* = 0.085) (Figure 1B). The corresponding incidence of 28-day mortality was 16% in the Adrecizumab group, vs. 25%, in the placebo group, *p* = 0.080 for difference.

**FIGURE 2:**
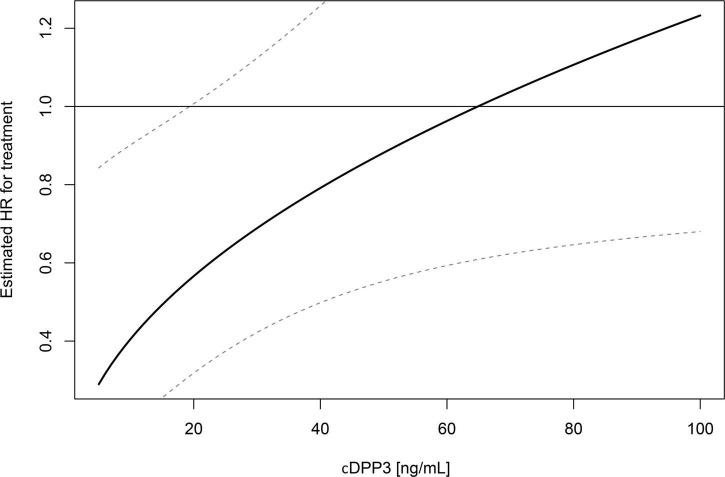
Hazard ratio (HR) of Adrecizumab treatment varying on circulating dipeptidyl peptidase 3 (cDPP3) level. Reported results are from an interaction analysis between Adrecizumab treatment and baseline cDPP3 concentration, i.e., a Cox regression was performed that included cDPP3, treatment status and the interaction term. HR and associated 95% CI compared to the placebo group are displayed.

To further assess the interplay between stratification based on cDPP3 and 28-day mortality, multivariable Cox proportional hazard modeling was performed. This multivariate model included cDPP3 (as a continuous variable), treatment group (Adrecizumab or Placebo), as well as the interaction term of both these variables. The model also included baseline SOFA scores as a correction for baseline disease severity. In this model, cDPP3, treatment and the cDPP3 * treatment interaction term were significantly associated with 28-day mortality; *p* = 0.015 for cDPP3, *p* = 0.017 for treatment group and *p* = 0.025 for the interaction term between cDPP3 and treatment group. An overview of the model’s associated hazard ratios and 95% CI’s is presented in Table 2. The hazard ratio of Adrecizumab treatment varying on cDPP3 level is displayed in Figure 2.

**TABLE 2:** Multivariate risk models of treatment interaction with circulating dipeptidyl peptidase 3 (cDPP3) (model 1) and time until start of treatment (model 2).

Variable	*n*	events	HR	95% CI	*P*-value
**Multivariate model 1**					
cDPP3	252	67	1.44	1.07–1.92	0.015
Treatment (Adrecizumab)	252	67	0.10	0.02–0.67	0.017
Baseline SOFA-score	252	67	3.51	1.30–9.49	0.014
cDPP3 * treatment (Adrecizumab)	252	67	1.72	1.07–2.77	0.025
**Multivariate model 2**					
TTT	254	67	0.97	0.46–2.04	0.932
Treatment (Adrecizumab)	254	67	0.37	0.04–3.71	0.397
Baseline SOFA-score	254	67	7.35	2.83–19.1	<0.001
TTT * treatment (Adrecizumab)	254	67	1.41	0.47–4.24	0.544

TTT, Time from diagnosis of septic shock until initiation of treatment (“Time till treatment”). * signifies the interaction term between two variables. Continuous variables [circulating dipeptidyl peptidase 3 (cDPP3), sequential organ failure assessment (SOFA)-scores, TTT] were log-transformed.

### Adrecizumab therapeutic efficacy by time to treatment after diagnosis of septic shock

**FIGURE 3:**
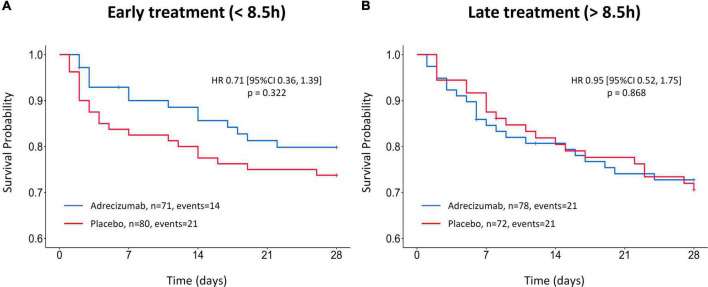
Kaplan–Meier of 28-day mortality after Adrecizumab/placebo infusion, based on treatment timing stratification. **(A)** Kaplan–Meier of patients treated <8.5 h after septic shock diagnoses. **(B)** Kaplan–Meier of patients treated >8.5 h after septic shock diagnosis. Both Adrecizumab dosing groups were combined. Crosses represent censored patients.

For the comparison of “early” and “late” start of Adrecizumab treatment, cohorts were first dichotomized at the median time from diagnosis of septic shock until start of treatment. Median [IQR] time from septic shock diagnosis to start of Adrecizumab treatment was 8.5 [5.9; 11.0] h. A total of 151 patients received treatment early after septic shock diagnosis (<8.5 h). The hazard ratio for 28-day mortality was not significantly lower for patients that were treated early (within 8.5 h) after septic shock diagnosis (HR 0.71 (95% CI 0.36–1.39) *p* = 0.322, *n* = 151, Figure 3A), compared to patients that started treatment later [HR 0.95 (95% CI 0.52–1.75) *p* = 0.868, *n* = 150, Figure 3B]. The corresponding incidence of 28-day mortality in the early treatment group was 20 vs. 26%, *p* = 0.342 for the Adrecizumab and placebo groups respectively, compared to 27 vs. 29%, *p* = 0.759 in the late treatment group. Next, a multivariable Cox proportional hazard model was generated, which included treatment timing (as a continuous variable), treatment group (Adrecizumab or Placebo) as well as the interaction term. This model also included baseline SOFA scores as a correction for baseline disease severity. In this model, both treatment timing and the interaction term were not significantly associated with 28-day mortality (Table 2).

### Adrecizumab therapeutic efficacy by circulating dipeptidyl peptidase 3 levels and treatment timing combined

**FIGURE 4:**
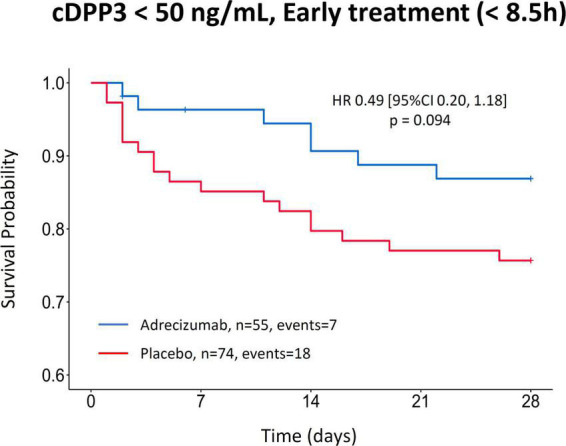
Kaplan–Meier of 28-day mortality after Adrecizumab/placebo infusion, based on a combined stratification approach implementing a cDPP3 <50 ng/mL as well as a treatment time <8.5 h after diagnosis of shock. Both Adrecizumab dosing groups were combined. Crosses represent censored patients.

The hazard ratio of 28-day mortality in a total of 129 patients with baseline cDPP3 levels <50 ng/mL and an early initiation of Adrecizumab treatment compared to patients that received placebo was HR 0.49 (95% CI 0.20–1.18), *p* = 0.094, (Figure 4). The corresponding 28-day mortality in this group was 13% for patients treated with Adrecizumab vs. 24% for patients treated with placebo, *p* = 0.099 for difference.

Multivariable Cox proportional hazard analysis through a model including both cDPP3, treatment timing and the respective interaction terms was not performed, as the interaction term associated with treatment timing was already not significantly associated with 28-day mortality.

### Associations between circulating dipeptidyl peptidase 3, treatment timing and sequential organ failure assessment score improvement after 24 h of treatment

**FIGURE 5:**
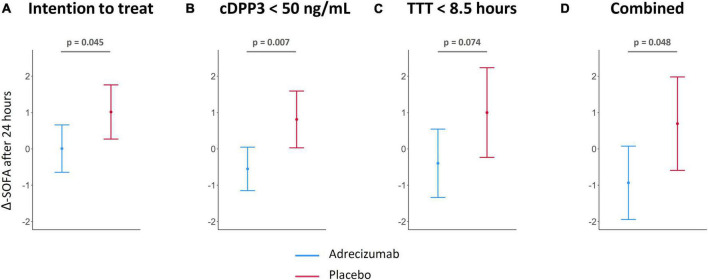
Change of sequential organ failure assessment (SOFA) score after Adrecizumab/placebo infusion, based on circulating dipeptidyl peptidase 3 (cDPP3) stratification. Data is displayed for the original intention to treat (ITT) analysis (15) **(A)**, the cDPP3 <50 ng/mL subgroup **(B)**, the early treatment (<8.5 h) subgroup **(C)** and the subgroup combining cDPP3 and treatment timing stratification **(D)**. The SOFA score was determined immediately prior to dosing and 24 h after start of treatment. The difference between the SOFA score pre-dosing and the SOFA score after 24 h of treatment was calculated for each patient, and the means ± 95% CI from the resulting values are represented in the Figure. For patients who died within 24 h of treatment, the SOFA was set to 24.

Change from baseline SOFA score 24 h after initiation of treatment (Δ-SOFA score) based on the different subgroup stratifications was investigated as an exploratory secondary outcome. In the ITT population, the Δ-SOFA score was more beneficial for the Adrecizumab group; being 0.01 point (95% CI −0.56; 0.58), vs. a further increase of 1.02 point (95% CI 0.35; 1.68) for the placebo group, *p* = 0.045 for difference (Figure 5A). When patients with a high baseline cDPP3 level (>50 ng/mL) were excluded, differences in Δ-SOFA scores became more pronounced, represented by a decrease of 0.55 point (95% CI −1.07; −0.03) vs. an increase of 0.81 point (95% CI 0.12; 1.50) in the placebo group, *p* = 0.007 for difference (Figure 5B). In contrast, patients treated with Adrecizumab with an earlier start of treatment (<8.5 h) did not display significantly more pronounced improvements in SOFA score after 24 h of treatment (Figure 5C). Correspondingly, a stratification approach combining cDPP3 and early treatment did not appear of more benefit compared to a stratification approach based on cDPP3 alone; SOFA score improvement of 0.93 point (95% CI −1.82; −0.05) in the Adrecizumab group vs. a deterioration trend of 0.70 point (95% CI −0.43; 1.82) in the placebo group, *p* = 0.048 for difference (Figure 5D).

### Adrecizumab safety by baseline circulating dipeptidyl peptidase 3 levels

No treatment related differences in adverse events (AE) and treatment emergent adverse event (TEAE) occurrence were found in the low cDPP3 subgroup; 96.5% AEs for Adrecizumab (combined dosage groups) vs. 94.0% AEs for placebo, *p* = 0.368 and 94.8% TEAEs for Adrecizumab (combined dosage groups) vs. 93.3% for placebo, *p* = 0.632.

## Discussion

In this *post-hoc* analysis of the AdrenOSS-2 trial, we demonstrated a significant interaction between cDPP3 levels and the therapeutic efficacy of Adrecizumab. In addition, we show that a shorter time to treatment initiation was not significantly associated with therapeutic efficacy. These results make it plausible that further enrichment of septic shock patient groups based on cDPPP3 concentration may increase chances of demonstrating therapeutic efficacy of Adrecizumab in future clinical trials, while a strategy aimed at further shortening time to initiation of treatment shows less promise.

Determining the cut-off of cDPP3 that allows for an optimal benefit-risk ratio for Adrecizumab treatment and, on the other hand, does not exclude an extensive group of sepsis patients that could still experience relevant benefit, goes beyond the data presented in this *post-hoc* analysis. Additional risk-benefit analyses performed during interim analysis of the phase-3 study of Adrecizumab should focus mainly on the impact of an imbalanced distributions of risks (demographics, critical parameters at baseline) on the results in subgroups of (even) smaller sample sizes that are no longer supported by randomized allocation.

Interestingly, in an observational study in septic shock patients, it was described that patients with high levels of both cDPP3 and bio-ADM had a substantially increased 28-day mortality compared to patients with high levels of only one of them (19). Although observational, this study first provided the first suggestion that bio-ADM and DPP3 might represent two distinct and partly independent pathophysiological mechanisms involved in the development of organ dysfunction during sepsis. In other words, an increased bio-ADM could be used to identify patients that may benefit from Adrecizumab therapy, whereas increased cDPP3 concentrations could be used to identify patients that may have lower chances of benefit, as they have an impaired prognosis based on another pathophysiological process that is hypothetically not influenced by Adrecizumab therapy.

The observed benefits of population enrichment with cDPP3 might be explained by the molecular pathways involved in the release of both bio-ADM and DPP3. DPP3 is a cytosolic enzyme, putatively reaching higher concentrations in the circulation in case of extensive cell injury (19, 26). During sepsis, this cell injury is likely caused by a profound impairment of microcirculatory tissue perfusion despite treatment. In animal studies, administration of exogeneous DPP3 provoked a rapid deterioration of left ventricular systolic function, putatively linked to reduced concentration of positive inotropic DPP3 substrates and/or increased concentration of negative inotropic products of DPP3 cleavage (27). In accordance with this finding, in preclinical models of sepsis and cardiogenic shock, inhibition of cDPP3 resulted in improved cardiac function index, as well as improved survival (21, 27). Interestingly, a cDPP3 blocking antibody is currently in preclinical stages of development (19, 27). During shock, upregulation of the renin angiotensin aldosterone system (RAAS) is a potentially life-saving response aimed at maintaining hemodynamic stability and adequate tissue perfusion (28). The degradation of compensatory RAAS response related mediators, e.g., angiotensin 2, caused by the uncontrolled release of DPP3 might be significantly contributing to the hemodynamic compromise in septic shock patients. As these mechanisms act independently of the ADM pathway, a more pronounced effect of Adrecizumab in patients without elevated cDPP3 levels appears plausible. Supporting this hypothesis, we demonstrated a significant interaction of cDPP3 levels with Adrecizumab treatment efficacy in a multivariate interaction analysis that was corrected for baseline disease severity.

The release of bio-ADM during sepsis represents a compensatory response aimed at restoring endothelial barrier function, a mechanism that falls short during refractory shock (5, 19). Observational studies have found a strong association of bio-ADM (at admission to ICU) with clinical outcomes in severe sepsis and septic shock (8–10). Additional studies showed that an even better prediction of outcome was provided by bio-ADM kinetics following the first 24 h of ICU treatment (10, 23, 24). Patients with high admission bio-ADM concentrations, that subsequently normalized after 24 h of treatment, demonstrated markedly improved chances of surviving (9.5% 28-day mortality), while patients in which bio-ADM concentrations remained high, or increased after 24 h, had a 28-day mortality of 38.1% (10). These results imply that adequate control of bio-ADM responses during the early hours of treatment of septic shock may relevantly impact outcome. In contrast, we found no significant associations between an early initiation of Adrecizumab treatment and Adrecizumab efficacy. Of note, the original AdrenOSS-2 study protocol already emphasized an early start of treatment after shock diagnosis, as only patients that could start study therapy within 12 h after start of vasopressors were eligible for study participation. Based on our results, it appears that this inclusion window of <12 h after shock diagnosis was already adequate to account for the early changes in bio-ADM responses mentioned above.

Our results have several research relevant implications. First, an upper limit of cDPP3 concentrations at baseline may serve as an additional patient enrichment criterion, allowing for the identification of a major subset of septic shock patients (with high bio-ADM levels but no elevated cDPP3 concentrations) that are more likely to benefit from Adrecizumab treatment. Second, while a timely start of Adrecizumab treatment after onset of septic shock might be of importance to improve chances of therapeutic benefit, our data do not support the necessity for an even earlier start of treatment than was achieved in the phase-2 trial. These findings emphasize the need for future sepsis trials to embrace enrichment strategies and, thereby, improve chances of detecting clinically relevant treatment effects in patient subsets that would otherwise be “diluted” in an unselected population. The upcoming phase III trial of the Adrecizumab program, called ENCOURAGE-1, aims to implement both strategies investigated in this *post-hoc* analysis, starting therapy early after initiation of vasopressor therapy as well as selecting patients based on rapid bedside assessment of relevant biomarkers. Based on our results, this trial will use a high concentration of bio-ADM as an inclusion criterium, while a high concentration of cDPP3 will serve as an exclusion criterium.

Despite the limitations of *post-hoc* analyses, the data presented here can provide relevant guidance for patient enrolment in future trials (e.g., the ENCOURAGE-1 trial). Further selection of subgroups results in smaller sample sizes, leading to limited statistical power. As the original study was a phase-2 safety and tolerability study, not powered to demonstrate beneficial effects on survival or other clinical endpoints, it is not surprising that several treatment differences in subgroups do not reach statistical significance. As for any *post-hoc* analysis, the results should be viewed as explorative and hypothesis-generating. Also, because cardiac function assessment, as well as measurements of hypoperfusion related parameters were not performed during the AdrenOSS-2 trial, the proposed pathophysiological mechanisms explaining the associations of cDPP3 with outcome remain largely speculative.

## Conclusion

Our results illustrate that a subset of septic shock patients with high bio-ADM levels also display high levels of cDPP3. We show that there is an interaction between cDPP3 concentrations and the therapeutic efficacy of Adrecizumab. This implies that increased efficacy of Adrecizumab may be present in the subgroup of patients with lower cDPP3 levels. Patient enrichment strategies implementing assessment of cDPP3 thus appear to identify a major subgroup of septic shock patients that may have an more pronounced benefit from treatment with Adrecizumab. The pathophysiological mechanism related to increased cDPP3 concentrations is putatively not related to bio-ADM and can, therefore, not be targeted by the treatment with Adrecizumab. These results reinforce the notion that from a pathophysiological point of view, sepsis should be viewed as a heterologous syndrome, rather than a single disease entity. To improve the chance of finding clinically relevant treatment effects, future sepsis trials should account for this heterogeneity by incorporating population enrichment strategies, preferably related to the mechanism of action of the therapy under investigation and/or exclusion of patients that show evidence of dysregulated processes that cannot be addressed by the intervention. These results warrant confirmation, which may be provided by ENCOURAGE-1, the phase III trial of Adrecizumab in septic shock patients, which will include cDPP3 assessment as well as early initiation of treatment as additional eligibility criteria.

## Data availability statement

The raw data supporting the conclusions of this article will be made available by the authors, without undue reservation.

## Ethics statement

The trial procedures and the informed consent form (ICF) process were approved by the respective independent ethics committee (IEC) following international standards and national requirements of each participating country. The AdrenOSS-2 trial was registered at Clinical trial.gov (NCT03085758). The patients/participants provided their written informed consent to participate in this study.

## AdrenOSS-2 study participants

Lila-Fariza Abeud, Pierre Asfar, Ludmila Baudrillart, Michael Bauer, Albertus Beishuizen, Caroline Berghe, Laure Berton, Paul Bourzeix, Diego Castanares, Kamile Cerlinskaite, Coralie Chalot, Benjamin Chousterman, Raphaël Clere-Jehl, Christine Collienne, Damien Contou, Thomas Daix, Julien Demiselle, Arnaud Desachy, Nicolas Deye, Tom Dormans, Anne-Aurore Duchambon, Thierry Dugernier, Marie-France Dujardin, Jacques Duranteau, Perrine Engels, Bruno Evrard, Anne-Laure Fedou, Marie-Celine Fournier, Bruno François, Alexandra Gay, Ludovic Gèrards, Leslie Gielens, Thomas Godet, Marine Goudelin, Tassadit Hadjam, Phillipe Hantson, Julie Helms, Isabelle Herafa, Oscar Hoiting, Alexa Hollinger, Vincent Huberlant, Tuija Javanainen, Philippe Jorens, Clement Jourdaine, Mahir Karakas, Stefan Kluge, Jean-Claude Lacherade, Jean-Baptiste Lascarrou, Matthieu Legrand, Badr Louadah, Martin Maëlle, Emmanuelle Mercier, Hamid Merdji, Ferhat Meziani, Alexandra Monnier, Virginie Montiel. Arthur Neuschwander, Haikel Oueslati, Gaëtan Plantefève, Julien Pottecher, Céline Prevost, Suzanne Renard, Tobias Schuerholz, Wytze Vermeijden, Philippe Vignon, Constance Vuillard, Emmanuel Weiss, Xavier Wittebole, Arthur van Zanten, and Alexander Zarbock.

## Author contributions

GM, P-FL, AM, and PP conceptualized the study. DL and AP drafted the manuscript. DL and OH performed the data analysis. KS performed the cDPP3 sample analyses and measurement quality control. All authors critically revised the manuscript and read and approved the final manuscript.

## Footnotes

1
http://www.r-project.org
