# Supplementary material for: Effects of enrichment strategies on outcome of adrecizumab treatment in septic shock: Post-hoc analyses of the phase II adrenomedullin and outcome in septic shock 2 trial
Source: Front Med (Lausanne). 2022 Dec 1;9:1058235. doi: 10.3389/fmed.2022.1058235 (PMC9751049; doi:10.3389/fmed.2022.1058235)
Supplement: Supplementary file 1 [file Table_1.DOCX]

***Supplementary table 1.*** *Demographics and baseline characteristics based on a cDPP3 cut-off of 50 ng/mL, as well as treatment with Adrecizumab or placebo. Differences in continuous variables were assessed with Kruskal-Wallis tests, while differences in categorical variables were assessed using Chi^2^ tests. Abbreviations: ITT = intention to treat, cDPP3 = circulating dipeptidyl peptidase 3, BMI = body mass index, SOFA = sequential organ failure assessment, APACHE = acute physiology and chronic health evaluation, HR = heart rate, MAP = mean arterial pressure, PCT = procalcitonin, eGFR = estimated glomerular filtration rate.*

| ITT | **cDPP3 <50 ng/mL** | | **p-value** | **cDPP3 >50 ng/mL** | | **p-value** |
| --- | --- | --- | --- | --- | --- | --- |
|  | **Treatment with placebo (n=134)** | **Treatment with Adrecizumab (n=115)** |  | **Treatment with placebo (n=18)** | **Treatment with Adrecizumab (n=31)** |  |
| Age (years) | 71.5 [60-78] | 71 [62-77] | 0.372 | 66.5 [61-72] | 70 [59-76] | 0.716 |
| BMI (kg/m2) | 26.54 [24.22-30.48] | 25.14 [23.12-29.48] | 0.049 | 26.61 [23.88-29.05] | 25.63 [23.44-28.07] | 0.534 |
| Gender, female, n (%) | 58 (43.28) | 40 (34.78) | 0.215 | 8 (44.44) | 11 (35.48) | 0.725 |
| **Severity scores** | | | | | | |
| SOFA (points) | 9 [7-11] | 9 [8-11] | 0.501 | 11 [9-14] | 11.5 [10-13.5] | 0.709 |
| APACHE II (points) | 32.5 [29-36] | 32 [29-34] | 0.455 | 33.5 [29-35] | 32 [27-38] | 0.554 |
| **Hospital admission characteristics** | | | | | | |
| Temperature (°C) | 37.1 [36.4-37.6] | 37 [36.2-37.7] | 0.886 | 37.05 [36.8-37.8] | 36.7 [36.3-37.4] | 0.130 |
| HR (bpm) | 96 [79-111] | 97 [83-112] | 0.622 | 97.5 [80-108] | 110 [93-128] | 0.093 |
| MAP (mmHg) | 72.5 [66-80] | 71 [66-78] | 0.676 | 70.5 [64-75] | 71 [65-79] | 0.500 |
| IL-6 (pg/mL) | 1687 [533.1-11162] | 2239 [429.4-10707] | 0.987 | 27048 [2407-112088] | 9146 [2657-39320] | 0.263 |
| PCT (ng/mL) | 34.01 [7.47-90.96] | 42.31 [8.77-86.58] | 0.646 | 79.89 [27.92-145.84] | 61.54 [27.87-120] | 0.419 |
| eGFR, MDRD (ml/min*1.73 m²) | 35.86 [21.28-51.09] | 33.97 [21.44-57.55] | 0.734 | 29.13 [20.91-35.41] | 28.37 [18.97-37.81] | 0.670 |
| Lactate (mmol/L) | 2.7 [1.5-4.5] | 2.9 [1.9-4.9] | 0.380 | 4.51 [3.57-8] | 5.52 [3.8-8.6] | 0.624 |
| PaO2/FiO2 (mmHg/%) | 271.33 [180-343.48] | 225.88 [172.34-337.65] | 0.283 | 203.97 [134-365.63] | 221.25 [143-327.14] | 1.000 |
| Fluid Input, first 24h (mL) | 2748 [1672-4985] | 2367 [1378-3934] | 0.112 | 3703.5 [2265.5-4765] | 4188.5 [3254-5039] | 0.272 |
| Time from septic shock to trt start  (h) | 8.33 [5.87-10.6] | 8.75 [5.58-11.08] | 0.356 | 10.1 [6.75-11.73] | 9.17 [5.67-11] | 0.217 |
| **Origin of Sepsis** |  |  | 0.766 |  |  | 0.125 |
| Lung, n (%) | 26 (19.4) | 27 (23.48) |  | 6 (33.33) | 3 (9.68) |  |
| Peritonitis, n (%) | 34 (25.37) | 24 (20.87) |  | 2 (11.11) | 5 (16.13) |  |
| Skin and soft tissue, n (%) | 12 (8.96) | 8 (6.96) |  | 2 (11.11) | 1 (3.23) |  |
| Urinary tract, n (%) | 24 (17.91) | 25 (21.74) |  | 2 (11.11) | 2 (6.45) |  |
| Other, n (%) | 38 (28.36) | 31 (26.96) |  | 6 (33.33) | 20 (64.52) |  |
